# Prediction of risk factors related to the development of hepatic dysfunction following open heart surgery

**DOI:** 10.1186/cc14353

**Published:** 2015-03-16

**Authors:** A Baysal, I Ozkaynak, M Dogukan

**Affiliations:** 1 Kartal Kouyolu Research and Training Hospital, Istanbul, Turkey; 2 Kahramanmaraþ Sütçü Ýmam University Hospital, Kahramanmaraþ, Turkey; 3 Adiyaman University Research and Training Hospital, Adiyaman, Turkey

## Introduction

Our goal was to investigate the incidence of postoperative jaundice in open heart surgery patients and to determine the risk factors associated with hepatic dysfunction.

## Methods

A total of 292 patients were included in a prospective study design. Patients undergoing on-pump coronary artery bypass graft surgery (CABG) (*n *= 154) and valve repair surgery (mitral, mitral and aortic valve and/or tricuspid valve) (*n *= 138) were included. Postoperative hyperbilirubinemia was defined as occurrence of a plasma total bilirubin concentration of more than 34 μmol/l (2 mg/dl) in any measurement during the postoperative period. All patients were divided into groups with or without hyperbilirubinemia. Liver enzymes were collected on postoperative days 1, 7, 14 and 30. The risk factors including age, cardiopulmonary bypass time, number of blood transfusions, inotropic support, use of intra-aortic balloon pump and ICU stay were evaluated with logistic regression.

## Results

Postoperative hyperbilirubinemia was observed in 27 of 292 patients (9.3%). The numbers of valves replaced, preoperative total bilirubin concentration, increased cardiopulmonary bypass time, higher number of inotropic support agents, and use of intra-aortic balloon pump correlate with hyperbilirubinemia on postoperative day 7 (*P *< 0.05). Independent risk factors of early postoperative jaundice are; multiple valve replacement surgery, ejection fraction and use of intraaortic balloon pump (*R *= 0.58, *R*^2 ^= 0.33, *F *= 26.44, *P *< 0.001). The ICU stay was significantly longer in group 2 (11.52 ± 3.76 days) as compared with group 1 (2.79 ± 1.36 days) (*P *< 0.001).

## Conclusion

Patients undergoing multiple valve replacement procedures are at greater risk for the development of postoperative hyperbilirubinemia and an association with prolonged ICU stay was observed. Other risk factors including ejection fraction, increased cardiopulmonary bypass time and use of intra-aortic balloon pump are also important as they have been reported to increase postoperative complications [1].
